# Associations of Compassion Competence, Nursing Work Environment, Organizational Communication Satisfaction, and Missed Nursing Care on Patient Safety Among Nurses in Comprehensive Nursing Care Service Wards: A Path Analysis

**DOI:** 10.3390/healthcare14172698

**Published:** 2026-08-24

**Authors:** SookHee Yoon

**Affiliations:** Department of Nursing, Semyung University, Jecheon-si 27136, Chungcheongbuk-do, Republic of Korea; top1490@semyung.ac.kr; Tel.: +82-43-649-1359

**Keywords:** compassion competence, work environment, communication, missed nursing care, patient safety

## Abstract

Background/Objectives: This study aims to identify the factors associated with patient safety for nurses working in comprehensive nursing care service wards, based on the Structure–Process–Outcome model. As comprehensive nursing care service wards expanded in South Korea, the number of older and more severely ill inpatients increased, raising the demand for nursing care. In these wards, nurses play a crucial role in ensuring patient safety. Therefore, a thorough identification of the factors associated with patient safety, based on theoretical frameworks, is necessary. Methods: A cross-sectional design was used. Data were collected from 379 nurses working in one general hospital and four tertiary hospitals in South Korea between March and April 2022. Data were analyzed through descriptive statistics, Pearson’s correlation coefficient, and path analysis using SPSS 26.0 and AMOS 28.0. Results: Missed nursing care (β = −0.152, 95% confidence interval [CI]: −0.276 to −0.039) and nursing work environment (β = 0.150, 95% CI: 0.017 to 0.298) showed a direct association with patient safety. Compassion competence (β = 0.040, 95% CI: 0.009 to 0.092) and nursing work environment (β = 0.027, 95% CI: 0.006 to 0.069) were indirectly associated with patient safety through missed nursing care. The variables accounted for 18.7% of the explanatory power for patient safety. Conclusions: Missed nursing care was the factor most strongly associated with patient safety. Therefore, organizational efforts to foster a positive nursing work environment and implement strategies to enhance nurses’ compassion competence may further contribute to patient safety.

## 1. Introduction

Patient safety represents a serious public health concern. Approximately 1 in 10 hospitalized patients suffer adverse effects during medical treatment, and patient harm ranks as the 14th leading cause of morbidity and mortality worldwide [1]. A study analyzing approximately 3 million official death records in the United States found that 0.16–1.13% of all deaths were caused by medical errors [2]. According to a report by the Korea Institute for Health Care Accreditation, adverse events, including sentinel incidents, accounted for 34.1% of the total 20,208 incidents in 2023 and 30.8% of the total 22,118 incidents in 2024 [3].

Comprehensive nursing care service wards are operated by a team of nursing professionals, without the need for family members or private caregivers, to provide safe and high-quality nursing care [4]. Since their introduction in 2015 with 105 healthcare institutions and 6945 beds, comprehensive nursing care service wards have expanded to 808 healthcare institutions and 87,105 beds by 2025 [5]. Unlike general wards in South Korea, where family members or privately hired caregivers have traditionally provided part of inpatient care, comprehensive nursing care service wards deliver all patient care through professional nursing staff and nursing assistants while maintaining higher mandatory nurse staffing levels than general wards [4]. Compared with general wards, these wards place greater emphasis on patient safety by requiring continuous monitoring and preventive nursing activities, including pressure ulcer prevention, fall prevention, infection control, and medication administration, all of which are key indicators in healthcare institution accreditation [6]. A study conducted among 346 nurses working in these wards found that 34.4% reported experiencing medical errors, with medication errors being the most common, followed by falls and errors related to procedures and treatments [7]. As patient acuity increases due to the growing number of older adults, patients with multiple comorbidities, and those requiring procedures or intensive monitoring, the demand for patient safety in comprehensive nursing care service wards rises as well [8]. Nurses provide continuous bedside care, playing a crucial role in ensuring patient safety by identifying potential risks and preventing adverse events [9]. Although the comprehensive nursing care service model is part of the South Korean health system, it reflects broader efforts to strengthen professional nursing care and improve patient safety—issues that are relevant across healthcare systems worldwide. Therefore, a thorough understanding of the factors associated with patient safety among nurses working in comprehensive nursing care service wards is needed to promote effective patient safety outcomes.

Compassion is a significant factor influencing patient safety [10]. The compassion competence of nurses is associated with their patient safety competency, playing a crucial role in safe nursing [11]. Positive nursing work environments [12], organizational communication satisfaction [13], and missed nursing care [14] are significant contributors to patient safety. In healthcare organizations, focusing on improving human factors and the organizational environment where nursing care is provided is essential to enhancing patient safety [10,15]. Despite the rapid expansion and operation of comprehensive nursing care service wards, few studies have taken an integrated approach to the factors related to patient safety among nurses. Therefore, this study explores how structural factors (nursing work environment, organizational communication satisfaction, and compassion competence) are associated with the care process (missed nursing care) and, ultimately, the outcome of patient safety.

Compassion is the ability to recognize another person’s suffering and make efforts to alleviate it. In nursing, compassion is considered a core professional competency and ethical foundation that improves patient outcomes by relieving suffering, enhancing treatment adherence, and promoting recovery [16]. Compassion competence refers to the ability to establish emotional connections and communicate with patients insightfully and sensitively [17]. Nurses need to quickly identify and respond to the increased demands of patients, and there is a high dependence on nursing staff [18]. Therefore, compassion competence is an essential professional qualification that enables nurses to recognize patients’ needs, respond appropriately to clinical changes in their conditions, and provide individualized, patient-centered care [19]. Nurses with higher compassion competence demonstrate higher levels of patient-centered nursing [20], and higher compassion competence is associated with the provision of higher-quality nursing services [21]. Nurses’ compassion competence has been found to be negatively correlated with the occurrence of missed nursing care [22], and compassion has been associated with better patient outcomes [23]. Research examining the relationships among compassion competence, missed nursing care, and patient safety remains limited. Therefore, this study examined the relationships among these variables.

The nursing work environment reflects the characteristics of healthcare organizations that support the provision of high-quality nursing care to patients. It encompasses overall support, including the physical environment, participation in decision-making, and securing autonomy, which can either support or impede professional nursing practice [24]. The perception of the nursing work environment in comprehensive nursing care service wards is generally more positive compared with that in general units [25]. A positive perception of the nursing work environment is a crucial factor in minimizing missed nursing care [26]. In other words, nurses working in more favorable nursing environments experienced a significantly lower likelihood and frequency of missed nursing care [27]. The nursing work environment, as perceived by clinical nurses, has been correlated with activities related to patient safety in nursing care [28]. Additionally, a better nursing work environment is associated with improved patient outcomes [12].

Organizational communication is the interaction between members to achieve the organization’s goals. In addition, it is a comprehensive emotional response to communication within the organization and refers to personal satisfaction [29]. Inadequate intra-team communication has been found to contribute to missed nursing care [30]. One study involving clinical nurses in Taiwan demonstrated that dissatisfaction with communication within the organization contributed to an increase in missed nursing care [31]. Additionally, research involving clinical nurses in Korea indicated that greater satisfaction with organizational communication had a positive effect on patient safety [13].

Missed nursing care refers to nursing care that is completely or partially omitted or delayed, including essential and common nursing care that should be provided to patients [32]. Throughout the COVID-19 pandemic, nurses faced new and challenging work conditions and unfamiliar diseases [33] and reported an increase in missed nursing care [34]. A study conducted on nurses working in comprehensive nursing care service wards in general hospitals found that 83% of the participants experienced missed nursing care [7]. Another study involving 1154 clinical nurses found that all participants reported at least one omission of nursing work during their most recent shift [35]. The occurrence of missed nursing care in a hospital setting increases the risk of negative outcomes, including adverse events, complications, disability, and patient mortality [36]. Missed nursing care not only poses a threat to patient safety [36] but is linked to a decline in the overall quality of nursing care, extended hospital stays, and increased readmission rates as well [37]. Previous studies have tested the theoretical relationship in which missed nursing care mediates the relationship between organizational factors and adverse patient safety events [38].

**Figure 1:**
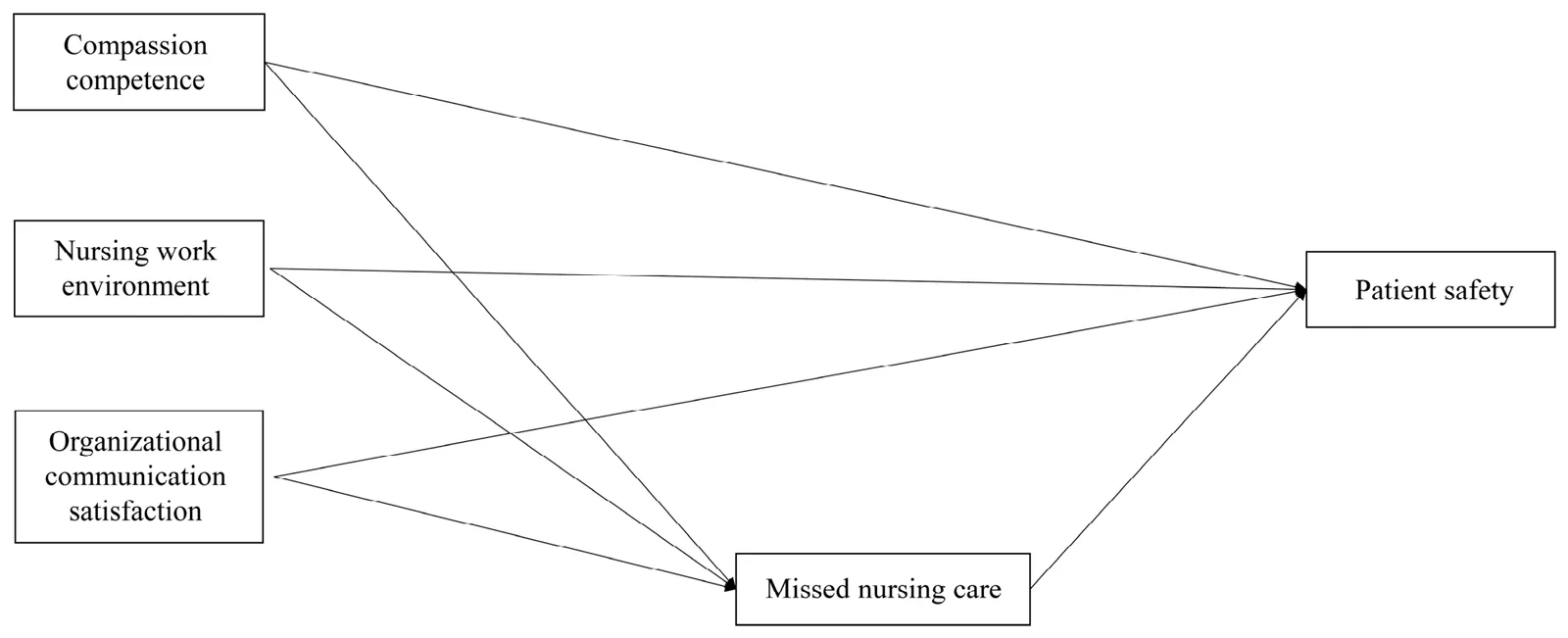
Hypothesized model.

A hypothetical model was developed, based on a literature review and the structure–process–outcome (SPO) model, to examine factors associated with patient safety. Donabedian’s SPO model is a theoretical framework that explains the quality of care in terms of structure, process, and outcome [39]. In this study, it was hypothesized that structural factors, including the nursing work environment, organizational communication satisfaction, and compassion competence, would have direct and indirect effects on patient safety through missed nursing care (Figure 1).

Research exploring patient safety factors in comprehensive nursing care service wards from a theoretical perspective is scarce. This study aims to contribute to establishing fundamental strategies for enhancing patient safety by comprehensively addressing these factors.

## 2. Materials and Methods

### 2.1. Design

This study employed a cross-sectional design and was reported in accordance with the Strengthening the Reporting of Observational Studies in Epidemiology statement.

### 2.2. Participants

Data were collected from nurses working in comprehensive nursing care service wards at one general hospital and four tertiary hospitals across multiple regions of South Korea. The participating hospitals were purposively selected because they had expanded comprehensive nursing care service wards and provided care for a high proportion of patients with severe illnesses requiring acute inpatient care. All participating hospitals operated comprehensive nursing care service wards according to national guidelines [40]. The participant selection criteria included nurses who had been employed in comprehensive nursing care service wards for over three months [7] and were directly responsible for nursing duties. Nurses or nursing managers who were not engaged in direct nursing duties in these wards were excluded. The sample size was based on the minimum required sample size of 200 for path analysis and the maximum likelihood estimation method [41]. The final analytic sample comprised 379 participants for the specific path model, which included 33 freely estimated parameters, among them those for the control variables. The sample also exceeded the recommended minimum participant-to-parameter ratio of 10:1 for path analysis [42] and was, therefore, considered adequate for the analysis.

### 2.3. Data Collection

This study was conducted following approval from the Institutional Review Board of the authors’ affiliated institution (No. X-4-2021-1762). The questionnaire was administered as an online survey. Data collection was conducted from March to April 2022. Participants were recruited from four tertiary hospitals and one general hospital, selected through convenience sampling. To ensure anonymity and encourage honest responses, participants’ hospital affiliations were not collected. This study was conducted after providing explanations to participants and obtaining their cooperation regarding the study plans, questionnaires, and methods. Recruitment notices, URLs, and QR codes were posted on the hospital websites and on the bulletin boards of the comprehensive nursing care service wards. Nurses who wished to participate in the study scanned the QR code posted on the recruitment notice or clicked the URL, which could be accessed online or through a mobile device. They read the study description, clicked the “I agree to participate in the survey,” and then participated in the survey. A total of 386 individuals responded to the online survey, and participants were offered a gift worth 30,000 KRW (approximately 25 USD in 2022) for their participation. After excluding the responses of seven individuals who answered insincerely, the data from 379 participants were analyzed.

### 2.4. Instruments

#### 2.4.1. Compassion Competence

Lee and Seomun [17] developed the Compassion Competence Scale for nurses. It comprises 17 items: 8 on communication, 5 on sensitivity, and 4 on insight. Each item was rated on a five-point Likert scale from 5 (“strongly agree”) to 1 (“strongly disagree”). A higher score indicates greater compassion competence in nurses. The overall Cronbach’s α of the scale was 0.91 in Lee and Seomun [17] and 0.90 in this study.

#### 2.4.2. Nursing Work Environment

Cho et al. [43] modified and adapted Lake’s [24] Nursing Work Environment Scale into Korean, which was used in this study. It comprises 29 questions. The subdomains are as follows: staffing and resources (4 questions), nurse manager ability, leadership, and support of nurses (4), collegial nurse–physician relationships (3), nursing foundation for quality of care (9), and nurse participation in hospital affairs (9). The statements about the nursing work environment were answered on a four-point scale ranging from 1 (“not at all so”) to 4 (“very much so”). Cronbach’s α of the scale was 0.82 in Lake [24], 0.93 in Cho et al. [43], and 0.93 in this study.

#### 2.4.3. Organizational Communication Satisfaction

Hong [44] modified Downs and Hazen’s [29] Organizational Communication Satisfaction scale to fit the Korean context. It comprises 24 questions: communication climate (5 questions), vertical communication (8 questions), horizontal communication (5 questions), and media quality (6 questions). Each question is rated on a five-point Likert scale ranging from 1 (“strongly disagree”) to 5 (“strongly agree”). The higher the score, the greater the level of organizational communication satisfaction. Cronbach’s α of the scale in Downs and Hazen [29] and Hong [44] was 0.90 and 0.90, respectively, and in this study, it was 0.89.

#### 2.4.4. Missed Nursing Care

Missed nursing care was evaluated using a tool translated into Korean and modified to suit the cultural context [45], based on the MISSCARE Survey developed by Kalisch and Williams [46]. The MISSCARE Survey comprises Part A and Part B. Part A includes 24 questions about elements of missed nursing care. Each question was evaluated on a four-point scale (1 = “rarely,” 2 = “occasionally,” 3 = “frequently,” and 4 = “always”). The reasons for missed nursing care (Part B) comprise 17 questions: labor resources (7 items), material resources (3 items), and communication (7 items). Each question was assessed on a four-point scale, with 1 representing “not a reason,” 2 “minor reason,” 3 “moderate reason,” and 4 “significant reason.” In Lee and Kalisch [45], Cronbach’s α was 0.90 for the element of missed nursing care and 0.90 for the reason for missed nursing care. In this study, Cronbach’s α values were 0.85 and 0.91, respectively.

#### 2.4.5. Patient Safety

Patient safety was assessed using a single item from the Patient Safety Culture survey developed by the Agency for Healthcare Research and Quality [47] and translated into Korean by Kim et al. [48], along with six items on adverse events derived from the nursing service quality tool initially developed by Purpora [49] and later translated into Korean by Lee and Kim [50]. The assessment of patient safety includes seven questions: patient safety grade (one item) and adverse events (six items). The patient safety level was measured on a five-point Likert scale, ranging from 1 (“very bad”) to 5 (“very good”). Adverse events were assessed using a five-point scale ranging from 0 to 4, where 0 = does not apply, 1 = never, 2 = rarely, 3 = occasionally, and 4 = frequently. Previous studies have demonstrated the validity of the patient safety grade [12,13]. For adverse events, the scores were calculated by reverse coding each question’s score. In the studies by Purpora [49] and Lee and Kim [50], Cronbach’s α values were 0.87 and 0.64, respectively. In this study, Cronbach’s α was 0.68.

### 2.5. Data Analysis

The data were analyzed using SPSS/WIN 26.0 and AMOS 28.0 (IBM Corp., Armonk, NY, USA). Descriptive statistical analysis was used to calculate the frequencies and averages of the sociodemographic and main variables. Pearson’s correlation coefficients were used to examine the relationships between the main variables, with no correlations exceeding 0.80, indicating the absence of multicollinearity. Univariate normality met the criteria, with absolute skewness values within 3 and absolute kurtosis values within 10 [41]. In addition, standardized residuals were approximately normally distributed; residual plots supported the assumptions of linearity and homoscedasticity; and tolerance (0.402–0.919), variance inflation factors (1.088–2.485), and Cook’s distance (maximum = 0.238) indicated no evidence of multicollinearity or influential outliers. Path analysis was conducted by specifying the main variables as observed variables using their mean scores, and missed nursing care was calculated as the average of Part A of the MISSCARE Survey. Age, total clinical experience [12], career in the present ward [7], and work pattern [51] were included as control variables in the path analysis based on the previous literature and preliminary analyses, as these variables showed significant differences or correlations with patient safety in the preliminary analyses. The maximum likelihood estimation method was used, and the significance of the direct, indirect, and total effects in the path model was assessed using the bootstrapping method. A total of 2000 resamples were used, and the significance of the effects was evaluated using bias-corrected 95% confidence intervals and two-tailed tests. The bootstrapped *p*-values were also reported [52]. The model fit was evaluated using several fit indices based on the following criteria: the root mean square error of approximation (RMSEA) and the standardized root mean square residual (SRMR) being under 0.08 [41], the chi-square to degrees of freedom ratio (χ^2^/*df*) being below 3 [42], and the Comparative Fit Index (CFI) exceeding 0.95 [53].

## 3. Results

### 3.1. Participant Characteristics

Most respondents (*n* = 367, 96.8%) were female, with an average age of 30.23 years (SD = 6.94). Among the participants, 272 (71.8%) were unmarried, and 86.8% held a Bachelor’s degree or higher. The average total clinical experience was 7.07 years (SD = 6.87), while clinical experience in the current ward was 3.72 years (SD = 3.38). A large proportion of the respondents—291 (76.8%)—were employed as staff nurses, and 351 (92.6%) worked in shifts. On average, nurses were assigned 7.87 patients (SD = 1.94). Over the past three months, 205 participants (54.1%) reported working overtime for more than 30 min but less than or equal to 60 min. Additionally, 360 respondents (95.0%) agreed that increased staffing is necessary in the nursing department (Table 1).

### 3.2. Descriptive Statistics of Variables

The average scores for the main variables were calculated, with patient safety, compassion competence, and organizational communication satisfaction having mean scores of 3.42 (SD = 0.44), 3.76 (SD = 0.48), and 3.01 (SD = 0.51) out of 5 points, respectively. The mean scores for the nursing work environment and missed nursing care were 2.42 (SD = 0.45) and 1.43 (SD = 0.28) out of 4 points, respectively (Table 2). Supplementary Table S1 shows that items with a missed nursing care frequency of over 50% include “attending interdisciplinary care conferences” (75.5%), “patient bathing/skincare” (67.3%), “emotional support for patients and/or family members” (64.4%), “ambulation three times per day or as ordered” (62.5%), “turning patient every 2 h” (55.7%), “full documentation of all necessary data” (52.2%), and “mouth care” (50.9%). The mean scores for the labor resources, material resources, and communication variables were 3.37 (SD = 0.48), 2.66 (SD = 0.76), and 2.48 (SD = 0.72) out of 4 points, respectively (Supplementary Table S1).

### 3.3. Correlation Between the Main Variables

Patient safety was positively correlated with compassion competence (*r* = 0.22, *p* < 0.001), nursing work environment (*r* = 0.32, *p* < 0.001), and organizational communication satisfaction (*r* = 0.29, *p* < 0.001) and was negatively correlated with missed nursing care (*r* = −0.19, *p* < 0.001). Missed nursing care was negatively correlated with compassion competence (*r* = −0.26, *p* < 0.001), nursing work environment (*r* = −0.14, *p* = 0.008), and organizational communication satisfaction (*r* = −0.10, *p* = 0.046; Table 3).

### 3.4. Path Analysis

**Figure 2:**
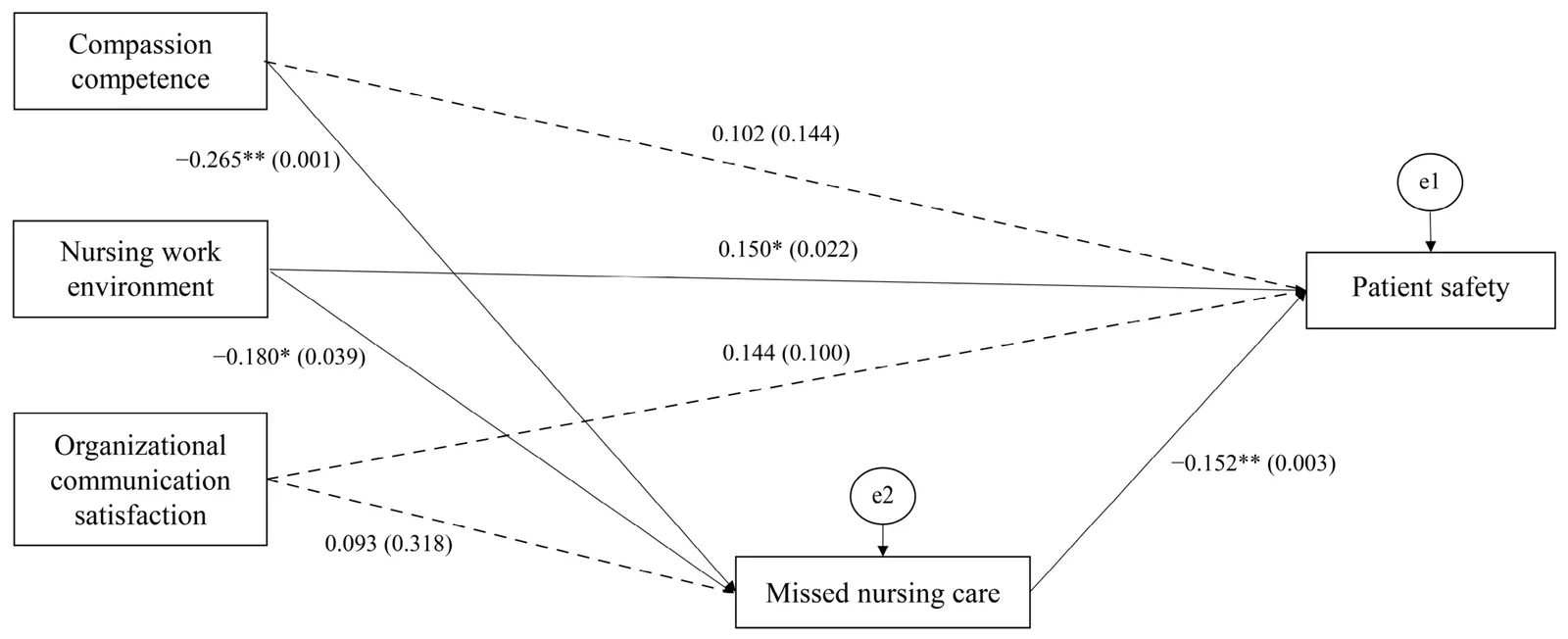
Path diagram of the model. Note. Standardized coefficients are shown for each path; The dashed lines with arrows represent non-significant relationships (*p* > 0.05). * *p* < 0.05; ** *p* < 0.01.

The standardized path coefficients obtained from the path analysis are presented in Figure 2 and Table 4. The model demonstrated a good fit (χ^2^/*df* = 2.643, *p* = 0.002; RMSEA = 0.066, 90% CI [0.038, 0.094]; CFI = 0.988; SRMR = 0.032). The control variables incorporated in the model included age, total clinical experience, length of career in the present ward, and work pattern. The analysis showed that compassion competence was not directly associated with patient safety but was indirectly associated with patient safety through missed nursing care (β = 0.040, 95% CI: 0.009 to 0.092, *p =* 0.003). The nursing work environment was directly related to patient safety (β = 0.150, 95% CI: 0.017 to 0.298, *p* = 0.022) and indirectly related to patient safety through missed nursing care (β = 0.027, 95% CI: 0.006 to 0.069, *p* = 0.014). Missed nursing care showed a negative association with patient safety (β = −0.152, 95% CI: −0.276 to −0.039, *p* = 0.003). Organizational communication satisfaction did not show a significant association with missed nursing care or patient safety. The explanatory power of the final model for patient safety was 18.7%.

## 4. Discussion

This study applied the SPO model to identify factors influencing patient safety using a sample of nurses working in comprehensive nursing care service wards. Unlike previous research that examined these factors in isolation, this study adopted a comprehensive approach by integrating factors (i.e., compassion competence, nursing work environment, and organizational communication satisfaction) and a process factor (i.e., missed nursing care) to analyze their relationships with patient safety. In addition, age, total clinical experience, length of career in the present ward, and work pattern were incorporated as control variables in the path analysis based on previous research. This approach enabled the relationships among the main study variables to be examined while accounting for potential demographic influences.

Nurses in this study reported average levels of perceived patient safety. Although direct comparisons are limited due to the scarcity of studies using the same assessment tools in comprehensive nursing care service wards, the current findings align with prior research conducted in general hospital wards [13]. These wards require a high level of nursing care, including bedsore prevention, medication safety, and infection control, as inpatients in these wards typically have more severe conditions and greater nursing needs than those in general wards [8]. Therefore, hospital management, including nurse managers, must enhance the working and physical environments to establish a comprehensive patient safety system [27]. Additionally, developing standard nursing practice guidelines and providing regular training are essential to equip nurses with the necessary skills as they take on new responsibilities in these wards [54].

In this study, missed nursing care showed a relatively stronger association with patient safety than the other variables examined, despite its modest effect size, consistent with previous research [14]. Among the seven most frequently missed nursing care activities, four were categorized as basic nursing—bathing/skin care, ambulation, turning patients, and oral care—which was similar to the findings of previous studies [45,55]. With the increasing severity of patients’ conditions upon admission to comprehensive nursing care service wards, recognition of the importance of tasks performed by professional nurses has grown, resulting in a heightened emphasis on direct nursing care that requires specialized knowledge and skills [54]. Since comprehensive nursing care service wards operate under a team nursing system—where nurses instruct and supervise nursing assistants and care support personnel—establishing practice guidelines that prioritize nursing tasks, ensure accurate delegation, and reduce missed nursing care is essential [4,40]. Further, inadequate staffing was identified as the primary cause of missed nursing care in this study, with most participants emphasizing the need for additional personnel. This finding is consistent with previous evidence demonstrating that staffing shortages substantially increase the likelihood of missed nursing care and compromise the delivery of essential nursing activities [55]. Accordingly, nursing managers, hospital administrators, and health policymakers must engage in discussions to secure adequate nursing staff, ensuring patient safety and the delivery of high-quality, patient-centered nursing services, given the severity of patient conditions and their nursing needs [36,55].

The findings suggest that the nursing work environment is positively associated with patient safety both directly and indirectly through missed nursing care, with a modest direct effect and a small indirect effect. These findings align with previous research indicating that a supportive nursing work environment is associated with a decrease in missed nursing care [56] and that nurses’ perceptions of their work environment are positively associated with patient safety [12,57]. At the organizational level, fostering a positive nursing work environment in these wards is crucial for ensuring patient safety and enhancing the quality of nursing care by minimizing missed nursing care. Therefore, diverse communication channels should be established to facilitate communication, information sharing, and collaboration among healthcare professionals. Additionally, nursing managers must establish appropriate staffing criteria based on a thorough analysis of ward units and encourage frontline nurses to participate in decision-making processes related to hospital policies and ward management [56].

The current findings also demonstrated that compassion competence was associated with lower levels of missed nursing care, which, in turn, was linked to improved patient safety through a small indirect effect. These results align with previous research indicating that nurses’ compassion competence is crucial in minimizing missed nursing care [22], whereas levels of missed nursing care are associated with an increased incidence of adverse patient safety outcomes [34]. Contrary to previous studies [23,58], no direct association was found between compassion competence and patient safety. This may be because patient safety is influenced by multiple individual and organizational factors, and the effect of compassion competence may operate through missed nursing care. Nurses with higher compassion competence may better recognize subtle changes in patient conditions and take timely action, thereby supporting the delivery of essential nursing care. Developing nurses’ compassion competence is essential for reducing missed nursing care and promoting quality care. Therefore, nursing organizations should not only provide education to strengthen individual nurses’ compassion competence but also create opportunities for nurses to share their clinical experience and support one another. These organizations should also foster an environment in which mutual caring and compassion are embedded in practice through sustained managerial attention and support [59].

Contrary to expectations, organizational communication satisfaction did not show a significant association with patient safety in this study. This finding differs from previous research, which found that nurses’ satisfaction with organizational communication shows a positive association with patient safety [13]. The lack of significant results for the role of organizational communication satisfaction among the examined organizational factors may be attributed to the relatively greater demands for patient safety activities (e.g., infection control and fall prevention) and the challenges of the nursing work environment (e.g., heavy workloads and staffing shortages) [6,60]. Additionally, organizational communication was not significantly associated with missed nursing care, which was inconsistent with previous findings [30]. This result may reflect the nature of comprehensive nursing care service wards, where direct information exchange and interpersonal interaction at the team level, rather than communication at the organizational level, are more practically relevant to nursing performance. Given the significant association between organizational communication and patient safety, further studies are needed to examine the relationship between various forms of communication and patient safety, including interactions between patients and nurses, as well as among different healthcare professionals, including physicians.

This study has several limitations. First, the use of self-reported data collected at a single time point may have increased the risk of common method and social desirability bias. Future studies should combine objective patient safety indicators with diverse data sources using mixed-methods approaches. Second, to preserve participant anonymity, data on affiliated medical institutions were not collected. Consequently, comparisons between tertiary and general hospitals were not feasible, which may limit the generalizability of the findings. Future studies should recruit participants from a wider range of healthcare institutions and account for institutional characteristics, including hospital type, bed capacity, and patient case severity. Third, the internal consistency of the adverse event measure was relatively modest (Cronbach’s α = 0.68). Although this level of reliability was considered minimally acceptable [61] and the patient safety measurement instrument was adapted from a previous study [13], future studies should further evaluate and refine the measurement of adverse events. In addition, future studies should examine the construct validity of patient safety measures that incorporate multiple dimensions of patient safety. Fourth, the path analysis supported the proposed mediation model, and the findings indicate statistical rather than causal mediation because of the cross-sectional study design. Longitudinal studies are needed to confirm the proposed mediation. Fifth, the study model explained only 18.7% of the variance in patient safety, suggesting that additional factors may influence patient safety. Future studies should incorporate additional factors not included in the present study, such as organizational culture, staffing levels, work-related factors, and system-level factors, to improve the model’s explanatory power and strengthen the evidence base. In addition, diverse methodologies, including qualitative and longitudinal studies, are warranted to strengthen the foundation for developing practical and evidence-based strategies to improve patient safety.

## 5. Conclusions

In this study, nurses working in comprehensive nursing care service wards identified missed nursing care as showing the strongest association with patient safety. A more favorable nursing work environment was directly and indirectly associated with patient safety through missed nursing care, whereas higher compassion competence was indirectly associated with patient safety through missed nursing care. These findings suggest that missed nursing care, the nursing work environment, and compassion competence are associated with patient safety, highlighting these factors as potential targets for future interventions and longitudinal research.

## Figures and Tables

**Table 1:** General characteristics (*N* = 379).

Variables	Categories	*N*	%	Mean (SD)
Sex				
	Female	367	96.8	
	Male	12	3.2	
Age (years)				30.23 (6.94)
	≤24	62	16.3	
	25–29	172	45.4	
	30–39	98	25.9	
	≥40	47	12.4	
Marital status				
	Single	272	71.8	
	Married	105	27.7	
	Other	2	0.5	
Education level				
	College degree	50	13.2	
	Bachelor’s degree or higher	329	86.8	
Total clinical experience (years)				7.07 (6.87)
	<1	32	8.4	
	1–<3	87	23.0	
	3–<5	78	20.6	
	≥5	182	48.0	
Length of career in present ward (years)				3.72 (3.38)
	<1	66	17.4	
	1–<3	127	33.5	
	3–<5	87	23.0	
	≥5	99	26.1	
Position				
	Staff nurse	291	76.8	
	Charge nurse	88	23.2	
Work pattern				
	Shifts	351	92.6	
	Fixed	28	7.4	
Number of assigned patients				7.87 (1.94)
Hours of overtime in the past 3 months				
	No overtime	6	1.6	
	≤30 min	77	20.3	
	>30 min ≤60 min	205	54.1	
	>60 min ≤120 min	75	19.8	
	>120 min	16	4.2	
Do you think the department needs additional staffing?				
	Yes	360	95.0	
	No	19	5.0	

Note. SD = standard deviation.

**Table 2:** Levels of compassion competence, nursing work environment, organizational communication satisfaction, missed nursing care, and patient safety (*N* = 379).

Variables	Range	Mean (SD)	Min	Max	Skewness	Kurtosis
Compassion competence	1–5	3.76 (0.48)	2.24	4.94	−0.07	0.02
Nursing work environment	1–4	2.42 (0.45)	1.00	3.76	−0.18	0.00
Organizational communication satisfaction	1–5	3.01 (0.51)	1.08	4.71	−0.24	0.75
Missed nursing care	1–4	1.43 (0.28)	1.00	3.54	1.70	8.46
Patient safety	1–5	3.42 (0.44)	1.92	4.50	−0.39	0.66

**Table 3:** Correlations between all variables (*N* = 379).

Variables	1	2	3	4
	r (p)	r (p)	r (p)	r (p)
1. Compassion competence	--			
2. Nursing work environment	0.16 (0.002)	--		
3. Organizational communication satisfaction	0.23 (<0.001)	0.77 (<0.001)	--	
4. Missed nursing care	−0.26 (<0.001)	−0.14 (0.008)	−0.10 (0.046)	--
5. Patient safety	0.22 (<0.001)	0.32 (<0.001)	0.29 (<0.001)	−0.19 (<0.001)

**Table 4:** Effects of predictor variables in the path model (*N* = 379).

Endogenous Variables	Exogenous Variables	Direct Effect	*p*	Indirect Effect	*p*	Total Effect	*p*	SMC
		β (95% CI)		β (95% CI)		β (95% CI)		
Missed nursing care	Compassion competence	−0.265 (−0.372, −0.154)	0.001			−0.265 (−0.372, 0.154)	0.001	0.109
	Nursing work environment	−0.180 (−0.348, −0.012)	0.039			−0.180 (−0.348, −0.012)	0.039	
	Organizational communication satisfaction	0.093 (−0.097, 0.272)	0.318			0.093 (−0.097, 0.272)	0.318	
Patient safety	Compassion competence	0.102 (−0.030, 0.236)	0.144	0.040(0.009, 0.092)	0.003	0.142 (0.014, 0.256)	0.029	0.187
	Nursing work environment	0.150 (0.017, 0.298)	0.022	0.027(0.006, 0.069)	0.014	0.177 (0.044, 0.326)	0.010	
	Organizational communication satisfaction	0.144 (−0.021, 0.286)	0.100	−0.014(−0.047, 0.011)	0.178	0.130 (−0.041, 0.270)	0.147	
	Missed nursing care	−0.152 (−0.276, −0.039)	0.003			−0.152 (−0.276, −0.039)	0.003	

Abbreviations: SMC, squared multiple correlation; χ^2^/*df* = 2.643 (*p =* 0.002), RMSEA = 0.066 (90% CI = 0.038, 0.094), CFI = 0.988, SRMR = 0.032; Control variables: Age, total clinical experience, length of career in present ward, work pattern.

## Data Availability

The original contributions presented in this study are included in the article/Supplementary Materials. Further inquiries can be directed to the corresponding author.

## Supplementary Materials

The following supporting information can be downloaded at: https://www.mdpi.com/article/10.3390/healthcare14172698/s1, Table S1: Missed nursing care (*N* = 379).

## Abbreviations

The following abbreviations are used in this manuscript:

RMSEA	Root mean squared error of approximation
SRMR	Standard root mean square residual
CFI	Comparative fit index
Min	Minimum
Max	Maximum
SD	Standard deviation
