# Supplementary material for: Associations of Compassion Competence, Nursing Work Environment, Organizational Communication Satisfaction, and Missed Nursing Care on Patient Safety Among Nurses in Comprehensive Nursing Care Service Wards: A Path Analysis
Source: Healthcare (Basel). 2026 Aug 24;14(17):2698. doi: 10.3390/healthcare14172698 (PMC13565695; doi:10.3390/healthcare14172698)
Supplement: Supplementary file 1 [file healthcare-14-02698-s001.zip › healthcare-4399326-supplementary.pdf]

Supplementary Table S1. Missed nursing care (N = 379)

Missed nursing care items	Frequency score	Prevalence of missed nursing care		
	Mean (SD)	n	%	Rank
**Part A [elements of missed nursing care]**	1.43 (0.28)			
Attending interdisciplinary care conferences	2.26 (0.95)	286	75.5	1
Patient bathing/skin care	1.89 (0.76)	255	67.3	2
Emotional support for patients and/or family members	1.82 (0.71)	244	64.4	3
Ambulation three times per day or as ordered	1.86 (0.81)	237	62.5	4
Turning patient every 2 hours	1.70 (0.72)	211	55.7	5
Full documentation of all necessary data	1.55 (0.56)	198	52.2	6
Mouth care	1.66 (0.74)	193	50.9	7
Assisting with toileting needs within five minutes of the request	1.50 (0.61)	166	43.8	8
Skin/wound care	1.42 (0.56)	150	39.6	9
Assessing the effective of medications	1.45 (0.62)	147	38.8	10
Teaching patient about procedures, tests, and other diagnostic studies	1.40 (0.57)	136	35.9	11
Setting up meals for patients who feed themselves	1.40 (0.72)	109	28.8	12
Hand washing	1.31 (0.52)	105	27.7	13
PRN medication requests acted on within 15 minutes	1.30 (0.51)	105	27.7	13
Focused reassessment according to patient condition	1.27 (0.47)	97	25.6	15
Medications administered within 30 minutes of the scheduled time	1.27 (0.48)	96	25.3	16
Feeding patient when the food is still warm	1.28 (0.56)	88	23.2	17
Response to call light initiated within five minutes	1.23 (0.47)	79	20.8	18
IV/central line site care and assessment according to hospital policy	1.18 (0.41)	66	17.4	19
Monitoring intake/output	1.14 (0.39)	47	12.4	20
Patient assessment performed during each shift	1.13 (0.39)	41	10.8	21
Vital signs assessed as ordered	1.11 (0.36)	35	9.2	22
Patient discharge planning and teaching	1.09 (0.30)	31	8.2	23
Bedside glucose monitoring as ordered	1.07 (0.29)	25	6.6	24
**Part B [reasons for missed nursing care]**	2.88 (0.55)			
Labor resources	3.37 (0.48)			
Material resources	2.66 (0.76)			
Communication	2.48 (0.72)			

Note: Prevalence of missed nursing care; Proportion of “always”, “frequently”, “occasionally” out of total responses
